# AI-driven biology: rethinking experiments and computation: An interview with Michael Bronstein, DeepMind Professor of Artificial Intelligence at the University of Oxford and Founding Scientific Director of the Aithyra Institute of the Austrian Academy of Sciences.

**DOI:** 10.1038/s44319-024-00268-6

**Published:** 2024-09-23

**Authors:** Thomas Lemberger

**Affiliations:** EMBO, Heidelberg, Germany

**Keywords:** Computational Biology, Science Policy & Publishing

## Abstract

Michael Bronstein outlines his vision for the new Aithyra Institute, which aims to transform biological sciences using AI, with a focus on developing novel approaches to data collection, model training, and hypothesis generation to advance research and improve human health.

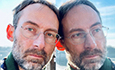

EMBO report (ER): Michael, it’s a great privilege to talk to you. We thought we can have a chat about fundamental research in machine learning and applications of AI in biology. Of course, we would like to learn more about the new institute you are about to lead in Vienna, and finish with your perspective on how AI may develop in the future.

AI has become popular everywhere and one of the inflection points was the release of chatGPT and the rise of large language models. A factor of their success is the ability to build so-called foundation models, which are trained on very large amounts of data and acquire broad generalization capabilities. Is there anything similar in biology, in drug design, and how do we get there?

**Figure:**
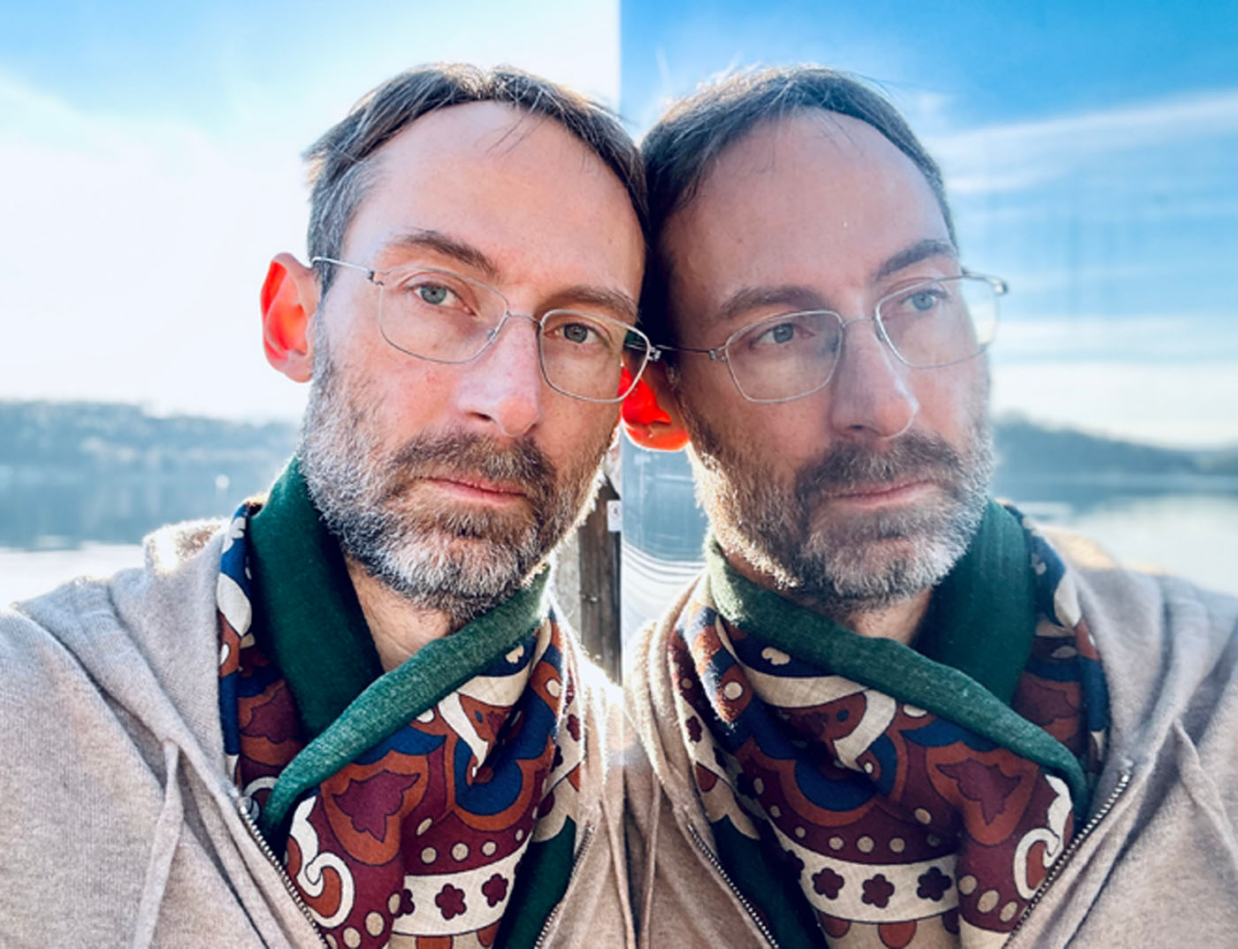
Michael Bronstein obtained his PhD from the Technion (Israel Institute of Technology) in 2007, where he studied problems at the intersection of geometry and machine learning. He is currently the DeepMind Professor of AI at the University of Oxford, where he develops geometric deep learning and generative models for molecular modeling and design. He received many awards with fancy names and has held appointments at places that traditionally top the university league tables. Michael is also a serial startupper and founder of multiple successful companies including Invision (acquired by Intel in 2012), Videocites, and Fabula AI (acquired by Twitter in 2019). He is Chief Scientist at the startup VantAI and advises multiple biotech companies including Recursion Pharmaceuticals and Relation Therapeutics. Aithyra is a new institute of the Austrian Academy of Sciences, made possible by a generous donation from the Boehringer Ingelheim Foundation. The leadership team includes Michael Bronstein as the Scientific Director and Anita Ender as the Administrative Director. AI was used to come up with the name of the new institute: Aithyra is the Greek goddess of biological AI, born from the union of Athena, the goddess of wisdom and strategy, and Asclepius, the god of medicine. Greek gods have traditionally accompanied the various disciplines of science; even in modernity they have never lost their importance as guardians, protectors, and sources of inspiration on a sometimes uncertain path into the future. Gabriella Sbordone

Michael Bronstein (MB): I think there is some broadness to the definition of what counts as a foundation model, and more generally, artificial intelligence. François Chollet posits that intelligence is not about how well a specialized model performs, but how good it is in learning new things, or in other words, how well it can generalize across tasks. In this sense we are probably still far away from “general intelligence,” and this is one of the reasons why I personally don’t like the term “artificial intelligence,” because we don’t really understand and agree on what intelligence is. In large language models such as ChatGPT, the key to their success was the scale, being able to create very large models that can be trained on huge amounts of data. We don’t yet have anything of comparable scale in biology, and probably the main limitation is the amount of data we have. It is very expensive to obtain experimental data. Maybe it will be possible to overcome the lack of experimental data with simulation and it’s an interesting question how to combine simulated data with experimental data. However, the range and diversity of problems in biology is significantly bigger than in language, and it could be that in some applications we don’t necessarily need the kind of scale we find in ChatGPT. There have recently been some publication claims about “foundation models,” for example in chemistry, but I think we are still a few years away from it.

“…the range and diversity of problems in biology is significantly bigger than in language, and it could be that in some applications we don’t necessarily need the kind of scale we find in ChatGPT.”

ER: Do you think there is a strategy to scale up the production of data in a form that is immediately usable by AI models, and at the scale necessary for training foundation models?

MB: Biotech and pharma companies try to do it and successfully in many cases. One example is Recursion that scaled-up cell-painting technologies, allowing to image hundreds of millions of cells and see what happens to the cells when you perturb them either chemically or genetically. What I would like to do is to take a step back and look at the next generation of data sources where the consumer of the data will not be a human but a machine. In a recent blog post that I co-authored with Luca Naef, my colleague and co-founder of the startup VantAI, we called it “black box data”. What we mean by this is a type of data that might not necessarily be understandable by a human and would only make sense in conjunction with appropriate machine-learning algorithms. AlphaFold, for example, was trained on PDB, a dataset collected by structural biologists for structural biologists. This data is very expensive and takes a lot of time to acquire—sometimes years for a single protein structure. If we say that the data does not necessarily need to be viewed by human scientists, we can come up with completely new experimental data sources, or maybe repurpose existing technologies that have never been considered suitable for a particular problem. For example, VantAI is using structural proteomics, which is up to 6 orders of magnitude cheaper than cryoEM, but doesn’t give the full structure of the molecules, only sparse interactions between them. But this is good enough with appropriate generative models to be able to generate new molecules, and we use such approaches to design molecular glues. This is something that has become possible only recently, when we had the right scale and the right machine-learning algorithms, like generative diffusion or flow matching. I think this conjunction of experimental technology and machine learning may potentially open an entirely new chapter in biological research.

“If we say that the data does not necessarily need to be viewed by human scientists, we can come up with completely new experimental data sources…”

ER: You are one of the pioneers in the field of geometric machine learning and have worked with graph neural networks. Why are these models so powerful? And what are the applications in biology and drug discovery?

MB: Geometric deep learning is based on a very old principle in science, which is symmetry and invariance. You can argue that, at some level, symmetry is the very foundation of science itself—everything from the structure of spacetime to the fundamental forces in the Universe can be derived from corresponding invariances and symmetries. What we try to do in geometric deep learning is to use similar principles to study and design neural network architectures. Historically, some of the deep-learning architectures that have become successful in computer vision and image analysis in the past decade, such as convolutional networks, were already based on such geometric ideas.

While these principles are well-known, I think it’s fair to say that only recently they have become more of a conscious design rather than just a happy coincidence. In the biological field, and especially in biochemistry, geometric architectures have become prominent maybe 5 years ago. You can represent molecules as graphs—nodes representing atoms linked by edges representing chemical bonds—you don’t care about how you order the atoms in the molecule, or the nodes in the graph. This is called permutation symmetry. For the prediction of many chemical and physical properties of the molecule, you also don’t care about how it is positioned in space. If, for example, you rotate or translate the molecule, its properties remain unchanged. Graph neural networks allow to incorporate such symmetries. The most prominent example of such an architecture is AlphaFold 2, and I think it has been an important factor that has contributed to the success of geometric approaches in biochemistry and the fact that these architectures are now widely used in drug design and drug discovery.

ER: Do you think that a synthetic-biology approach could be useful to explore protein design space? For example, synthesize potential candidate protein domains, measure their biochemical characteristics and then improve models in cycles of training and new experiments?

MB: I would say that we should use all the data that is possible, and another avenue is simulation. As far as I know, companies such as Isomorphic Labs are working on simulated data and accelerating or even potentially replacing expensive simulations like molecular dynamics with generative machine learning models. This is another interesting avenue that we would like to explore in the new institute, which, if successful, might bring a profound change to the ways that computational science is done. If you look at areas from weather forecasting and climate modeling to molecular simulations, they rely on computational models rooted in small-scale laws that we know and understand well and that we try to apply at large scales where the governing laws are much less understood. Such simulations are usually very computationally intensive and require fine spatial and temporal resolution to work well. It might be possible to accelerate or even entirely bypass numerical simulations altogether with appropriate generative machine-learning models. It would be very interesting, because it will probably be for the first time that we’ll forego mathematically- or physically-principled models for something that is significantly faster and scalable but allows for mistakes in certain cases but overall makes sense.

“It might be possible to accelerate or even entirely bypass numerical simulations altogether with appropriate generative machine-learning models.”

ER: Speaking of the new institute that you will be heading, tell us more about the key idea behind this initiative.

MB: My ambition is to make Aithyra a cool new and magical place in central Europe. That’s something that doesn’t happen every day. We would like to transform the way biological sciences are done with the help of AI, in order to drive the biological revolution in the next decade with the goal of ultimately improving human health. We want our research to have a tangible impact on diagnosing and curing diseases, developing new drugs, understanding better how biological systems work in our body and how they stop working when we get sick. We want to co-locate biologists and computer scientists in the same place so we learn from each other and work on the next generation of machine learning and experimental technologies together. In my view, this is a necessary condition for the next breakthrough. It’s hard to tell now where these breakthroughs might happen, so at this point we are open-minded about what biological problems we want to consider. But we would like to consider biological problems from the perspective of machine learning, so we will likely favor problems in which it is possible to collect large amounts of data and where the problem itself is likely to be amenable to machine-learning solutions.

“We would like to transform the way biological sciences are done with the help of AI, in order to drive the biological revolution in the next decade with the goal of ultimately improving human health.”

ER: In the organization of an institute like this, will you try to combine data production and model training? Do you have some ideas how to structure the institute, such that it is tailored to AI and machine learning?

MB: Absolutely so. We would like to create a robotic lab, so we can test machine learning algorithms in the loop to produce and consume the data. We would also like to improve heavy simulations with the help of machine learning and use simulated data to train machine-learning systems. Everything will be released in the public domain; we would like to contribute to the community by publishing code, models, and data.

ER: In terms of computing scale for training, is that an issue? What access do you have to GPU power to train all these models?

MB: Some of the models are indeed very compute intense, but in different ways. Large-scale transformers, such as those used in LLMs applied for example on genomics data are GPU-hungry, whereas other problems such as traditional physics simulations mostly rely on CPUs. We certainly would like to be one of the better equipped places in Europe in terms of compute power and budget significant cost for computer infrastructure.

ER: Do you plan to have strong private-academic partnerships, or how do you see that sort of connection to translational applications?

MB: The mandate of the institute is pure research. It will be an institute of the Austrian Academy of Sciences, and a significant amount of research funding, €150 Mio. over a period of 12 years, will be provided by the Boehringer Ingelheim Foundation, as a nonprofit institution. Having said that, I would like to create a startup and venture capital ecosystem around it, because some of the research that we’ll be producing will likely be commercializable. We would like our future researchers and students to take their ideas out and make an impact in the broader industry. My aspiration would be to make it the friendliest place for startups in Europe to translate research discoveries into impactful solutions for society.

ER: There’s often a tension between the private and the academic world. We’ve seen that with AlphaFold 3: it was released and published but the code was not available, and part of the community reacted strongly to this. What are your views? Should all models be immediately open source, or is there some room for keeping them proprietary for a while?

“One of the reasons why the machine learning community has made such a rapid progress was because most of the research was open-sourced.”

MB: One of the reasons why the machine learning community has made such a rapid progress was because most of the research was open-sourced. We definitely would like to adopt the same mindset. We would also like to collaborate with industry, but I think the majority of the work we’ll be doing will contribute to the academic research community.

ER: You mentioned that scale and the amount of data are a determinant criterion for many applications and models. In the academic world, do we have access to enough computing power to reach this scale?

MB: In terms of access to computing power, I wouldn’t make general statements, because I think the problems in machine learning can vary a lot depending on how computer-hungry they are. For some applications like LLMs, academia is absolutely out of the race with industry. Just to give you an example, Princeton prides itself with a cluster of something like 300 GPUs, and one of the biggest clusters in the biotech industry is Recursion with more than 500. Meta alone has hundreds of thousands and will soon surpass half a million GPUs. Universities obviously cannot compete with leading big tech companies and should rather be working on other problems that might not require immense computational resources, and I believe there are plenty of such problems in biology. The more compute-hungry problems, might need to be solved by collaboration with industry.

ER: As a fresh institute, you will have to recruit new talents. What are the skills that are necessary these days for young scientists to be ready for AI in biology?

MB: Compared to a few years ago, we see increasingly more computer scientists that are interested in biological problems. And likewise, there are many biologists who are interested in using machine learning in their field. It’s very good that there is this percolation. In the new Institute, I would expect machine learners to at least have a broad understanding of biology, maybe a deeper understanding of the specific problems they are working on, but not necessarily be able to produce new results in this domain on their own. In the same way, a biologist would be knowledgeable about how machine learning works and would be able to use it, but not necessarily be able to develop new algorithms. It’s important to put them together in the same building so they breathe the same air and dream the same dreams. We need to offer an attractive work environment. We would like to be the best employer fostering an inclusive and diverse culture investing and taking care of our people.

ER: Your background is in mathematics or computer science?

MB: Computer science, yes.

ER: Did you find it difficult to understand biology and to think about these problems?

Michael Bronstein: I would not claim that I understand biology, but I also don’t think I would call it “difficult”—“exciting” is probably a better term. My first biological machine-learning collaboration was with Bruno Correia, a structural biologist based at EPFL in Switzerland. These were very cool problems with a lot of geometry. I find that when a newbie comes to your field and starts asking silly questions, in most cases the answer is trivial, but in other cases, you actually start thinking, “why are we doing it this way and not another way,” and when you cannot explain it to yourself, you may come to the conclusion that you can do it better or completely differently. It happened to me a couple of times and it was very rewarding. This is the kind of interactions I would like to have between biologists and computer scientists.

ER: We are all excited about AI, and it’s a profound change even in how to think about biology and new experiments. But it also raises some concerns about the power of these methods, their reliability, potential misuses, and the growing autonomy of AI systems. What should the research community do to develop AI in a responsible way that keeps the trust of decision-makers and the public in general?

MB: There are many concerns about applications where AI replaces humans or AI makes decisions that impact human life, be it the recommendation algorithms on social media, or AI-assisted decisions that are made by governments, the judicial system or financial institutions. I think regulation is probably inevitable to ensure that such systems are used fairly and to the benefit of the society, but I’m also concerned about over-regulating the field. We don’t want to choke it while it is still nascent. In the life sciences, it is possible that we are going towards a new way of doing science with the help of AI. Probably for the first time in the history of the scientific method, humans might be augmented and perhaps, in a more remote future, even replaced by machines in the process of generating new hypotheses, which has so far been a hallmark of human ingenuity and creativity. This is something that we would like to look into: can we generate new theories or designs new experiments with the help of AI, basically making automated scientists?

“Probably for the first time in the history of the scientific method, the part of hypothesis generation, that has been the hallmark of human ingenuity and creativity, might be augmented and perhaps, in a more remote future, even replaced by machines.”

From a philosophical perspective, it poses big questions of how science will look like when it’s not done by humans. There are proponents of a “post-theory science,” when we have theory that cannot be written in the form of equations that we, humans, understand. Instead, it’s a black box with many parameters that are tuned on the data, but it produces predictions that can be tested experimentally. AlphaFold is a good example. It does not offer a “theory of protein folding,” but it produces correct predictions that are useful in many situations.

One of the concerns raised about biological AI is biosafety. My main concern would not be the ability of AI to design toxic compounds—we have plenty of very toxic stuff already without any AI, and the challenge in drug design is how to make it selectively toxic—but harmful organisms and viruses. At this point in time, it is a possible but, in my view, still somewhat remote scenario. However, since the field evolves very fast, we might transition into this more harmful reality faster than we anticipate, so it’s very important to be prepared for it.

ER: You mentioned the autonomy of living organism. Now with AI, we reach a high level of autonomy and there is a strong motivation to push this even further to generate more data and hypotheses. Do you think we should put some cap on this autonomy? Or should we let self-reproducing robots do drug screening on our behalf?

MB: We don’t really have self-reproducing AI, and in general it works differently from biological organisms. In my opinion, we should not see AI as some kind of uncontrolled force of nature. Rather, it’s a technology that we design and can shape in a way that is useful for humankind. Achieving this requires scientists doing the right things, as well as political effort and regulation. You might call me a technological positivist, but I don’t see the AI doomsday scenarios as unavoidable or even likely.

ER: For the future what might be the next critical technological or theoretical advance in AI that will bring it to the next level?

MB: I would say, a big achievement in AI for science would be accelerating or replacing traditional and notoriously heavy numerical simulations like molecular dynamics or fluid dynamics with generative machine learning models trained on experimental or simulated data. There are still many open questions, but if successful, the impact could be tremendous because such models appear across the board in computational sciences.

ER: You have worked on many different topics, and you are a serial entrepreneur. You were Head of Graph Learning at Twitter. You also mentioned a couple of companies you are involved in now. You even published a paper on social communication between whales. And now you will be the head of the new institute. How do you keep up with all of this?

MB: Well, I am lucky to have had amazing students and collaborators. Science is a collective effort and I’ve had the chance to work with the best people in their respective domains. I intend to keep it this way and bring the best talents under the same roof of the new institute. Biology is a very broad field, with a lot of exciting problems, and the AI and machine learning research community has a special culture allowing the field to move very fast. What we are working on now didn’t exist a few years ago. Even to people in the field, the capabilities we see today, just a decade ago would have seemed science fiction. We want to bring in some of this culture and speed into the more traditional science.

ER: Professor Bronstein, thank you for the interview.

The interview was conducted by Thomas Lemberger, Head of Open Science at EMBO.

